# Macrocyclic Chelates Bridged by a Diaza-Crown Ether: Towards Multinuclear Bimodal Molecular Imaging Probes

**DOI:** 10.3390/molecules25215019

**Published:** 2020-10-29

**Authors:** Gaoji Wang, Goran Angelovski

**Affiliations:** 1 MR Neuroimaging Agents, Max Planck Institute for Biological Cybernetics, Max-Planck-Ring 11, 72076 Tübingen, Germany; gaoji.wang@tuebingen.mpg.de; 2 Lab of Molecular and Cellular Neuroimaging, International Center for Primate Brain Research (ICPBR), Center for Excellence in Brain Science and Intelligence Technology (CEBSIT), Chinese Academy of Science (CAS), Shanghai 200031, China

**Keywords:** diaza-crown ether, hetero-multinuclear complexes, lanthanide, luminescence, macrocyclic, paraCEST, water exchange

## Abstract

Bridged polymacrocyclic ligands featured by structurally different cages offer the possibility of coordinating multiple trivalent lanthanide ions, giving rise to the exploitation of their different physicochemical properties, e.g., multimodal detection for molecular imaging purposes. Intrigued by the complementary properties of optical and MR-based image capturing modalities, we report the synthesis and characterization of the polymetallic Ln(III)-based chelate comprised of two DOTA-amide-based ligands (DOTA—1,4,7,10-tetraazacyclododecane-1,4,7,10-tetraacetic acid) bridged via 1,10-diaza-18-crown-6 (DA18C6) motif. The DOTA-amide moieties and the DA18C6 were used to chelate two Eu(III) ions and one Tb(III) ion, respectively, resulting in a multinuclear heterometallic complex **Eu_2_LTb**. The bimetallic complex without Tb(III), **Eu_2_L**, displayed a strong paramagnetic chemical exchange saturation transfer (paraCEST) effect. Notably, the luminescence spectra of **Eu_2_LTb** featured mixed emission including the characteristic bands of Eu(III) and Tb(III). The advantageous features of the complex **Eu_2_LTb** opens new possibilities for the future design of bimodal probes and their potential applicability in CEST MR and optical imaging.

## 1. Introduction

In modern medicine, magnetic resonance imaging (MRI) is recognized as the method of choice for non-radiative and non-invasive imaging of soft body tissues [1,2,3]. Conventional MRI exploits the magnetic properties of water proton’s spins to generate signal. However, it suffers from intrinsic insensitivity, which often requires the administration of contrast agents (CAs). Specifically, these mainly paramagnetic Ln(III)-based complexes improve specificity of the MRI scans through various contrast-generating mechanisms [3]. One of the recently introduced MRI strategies is based on the chemical exchange saturation transfer (CEST) effect, which possesses specific advantages over the conventional *T*_1_-weighted MRI. Namely, the CEST MRI enables generation of the signal at will by using selected radiofrequency (RF) pre-saturation pulses, thus providing improved specificity in these MRI studies due to the absence of permanent background signal originating from the CEST agent [4,5]. The principle of CEST detection is based on the selective saturation of the pool of protons that are in slow to intermediate exchange rate on the NMR time-scale, with bulk water pool. Subsequently, the chemical exchange of the pre-saturated CEST proton pool with the water protons reduces the intensity of the MR signal at the frequency of the bulk water protons. The general requirement for successful CEST is that the frequency difference between the two pools of protons (Δω) is greater than the corresponding exchange rate (*k*_ex_) [6]. Moreover, the paramagnetic lanthanide(III) ions, such as Eu(III), Tb(III) or Yb(III), are known to induce chemical shifts (and hence Δω), which makes them perfect candidates for paramagnetic CEST (paraCEST) CAs [7,8,9]. As a general rule, the CEST effect is induced either by the exchangeable proton of the ligand or by the water molecule coordinated to Ln(III). Typically, Ln(III) complexes based on DOTAM (DOTA-tetraamide, DOTA—1,4,7,10-tetraazacyclododecane-1,4,7,10-tetraacetic acid) are characterized by an exchange rate suitable for CEST imaging [6]. Although the thermodynamic stability of the Ln(III)-DOTA-tetraamide complexes is considerably lower than that of the tetracarboxylate analogues, their kinetic inertness is extremely high, which makes them good candidates for potential in vivo applications [3,10,11,12].

Furthermore, Ln(III) ions, especially Eu(III) and Tb(III), have often been employed for optical sensing owing to their specific sharp line-like emission bands and long lifetimes, often being in the milliseconds range [13]. These features allow implementation of the time-gated techniques to increase the signal-to-noise ratio (SNR). Most importantly, lanthanide luminescence has large Stokes shifts (>200 nm) when compared to typical organic fluorescence compounds. Such unique photophysical properties circumvent the autofluorescence, as well as the light scattering from backgrounds of the biological samples, such as cell cultures or tissues [14]. Thus, besides suitable properties for optical imaging, complexes comprised of two different Ln(III) ions might provide properties suitable for dual-modal imaging, enabling the design of dual- and multi-modal probes [15,16,17,18,19,20,21].

**Chart 1:**
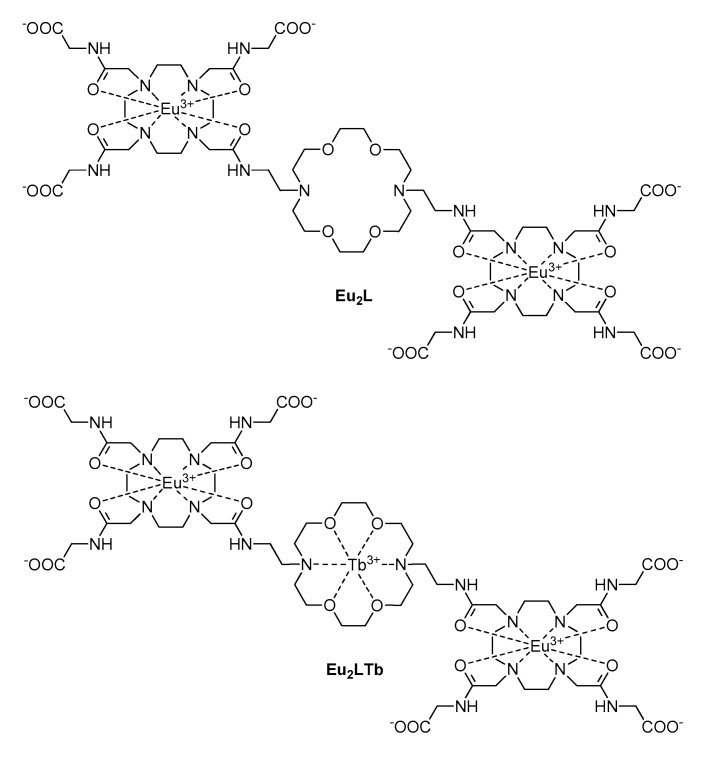
The chemical structures of **Eu_2_L** and **Eu_2_LTb** investigated in this work.

In this work, we set out to investigate the possibility of developing a dual-modal agent that is suitable for CEST MR and optical imaging (Chart 1). We designed a molecule that consisted of two DOTAM-Gly macrocyclic moieties and a DA18C6 (DA18C6- 1,10-diaza-18-crown-6) azacrown moiety, intended for chelation of two different paramagnetic and luminescent Ln(III) ions. The DOTAM-Gly accommodated two Eu(III), giving rise to strong paraCEST signal of the generated dinuclear complex. The interaction of Tb(III) with the azacrown moiety resulted in the increase of the Tb-centered luminescence emission, showing the advantageous properties of the prepared multinuclear heterometallic complex as a potential dual-modal imaging probe.

## 2. Results and Discussion

### 2.1. Synthesis of Complexes Eu_2_L and Eu_2_LTb

**Scheme 1:**
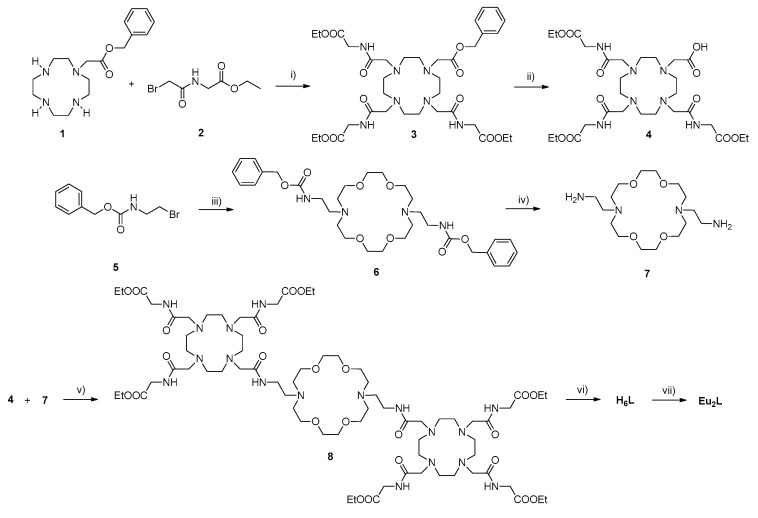
Synthesis scheme of complex **Eu_2_L**. Reagents and conditions: (**i**) Na_2_CO_3_, DCM, r.t. 12 h; (**ii**) H_2_/Pt, DMF, r.t., 12 h; (**iii**) MeCN, Cs_2_CO_3_, 65 °C, 4.5 h; (**iv**) H_2_/Pt, EtOH, r.t., 4 h; (**v**) HATU, DMF, r.t., 6 h; (**vi**) LiOH, MeOH, r.t., 12 h; (**vii**) EuCl_3_∙6H_2_O/H_2_O, 50 °C, 12 h.

The desired trismacrocyclic ligand was synthesized in a stepwise manner, using glycine ethyl ester hydrochloride, bromoacetyl bromide, 2-bromoethylamine hydrogen bromide and benzyl chloroformate as starting compounds (Scheme 1). In the first step, the cyclic polyamine **1** was alkylated with three equiv. of the bromide **2** to provide the macrocycle **3**. Hydrogenation of **3** catalyzed by Pd/C in DMF afforded the acid **4** in high yield. In the second, azacrown fragment **7** was prepared in a three-step procedure. The commercially available 2-bromoethylamine hydrogen bromide was reacted with benzyl chloroformate to yield Cbz-protected amine **5** [22]. Then, two equiv. of **5** were reacted with 1,10-iaza-18-crown-6 to acquire azacrown dicarbamate **6**, which was then subjected to the catalytic deprotection of Cbz groups by hydrogenation in the presence of Pd/C in ethanol to afford the diamine **7**. Coupling of two equiv. of the acid **4** with the diamine **7** resulted in the thismacrocyclic precursor **8**. Finally, the base hydrolysis of **8** was achieved with LiOH to give the final ligand **H_6_L**. The obtained compounds were characterized by ^1^H/^13^C-NMR and mass spectrometry. The bimetallic complex **Eu_2_L** was prepared by treating the final ligand **H_6_L** with EuCl_3_∙6H_2_O in water, while maintaining the pH at ~7. Subsequently, complex **Eu_2_LTb** was prepared by treating **Eu_2_L** with one equiv. of TbCl_3_ aqueous solution and was characterized by LC-MS spectrometry.

### 2.2. CEST Effect Measurements of **Eu_2_L**

**Figure 1:**
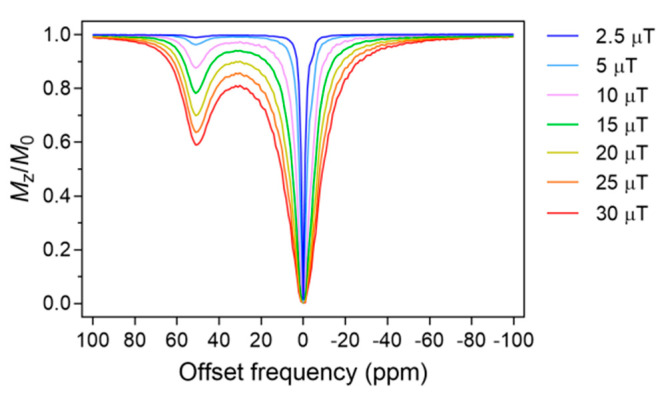
The CEST spectra of the complex **Eu_2_L** (5 mM, irradiation time 10 s, in 50 mM HEPES with pH 7.4, 25 °C) recorded at different *B*_1_.

Given that the EuDOTAM-Gly complex is a well-studied paraCEST agent, the CEST properties of its structural analogue **Eu_2_L** were investigated in detail and compared accordingly [23,24,25]. The CEST spectra of 5 mM **Eu_2_L** were recorded with 10 s irradiation time and variable saturation powers (*B*_1_ = 2.5, 5, 10, 15, 20, 25 and 30 μT) at 25 °C and pH 7.4 (Figure 1). The CEST signal resonating at ~50 ppm was observed for **Eu_2_L**, corresponding to the proton exchange between Eu(III)-bound water and the bulk water molecules. The CEST effect depends on the saturation power variation [26], resulting in a CEST effect increase from ~5% to ~60% for the increase in saturation power from 5 μT to 30 μT, respectively (Figures S1 and S2, Supplementary Materials).

**Figure 2:**
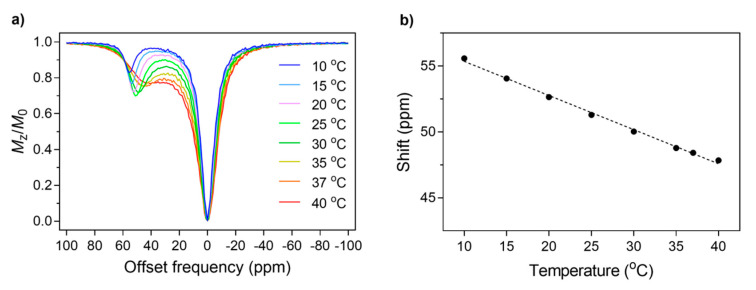
(**a**) The CEST spectra of 5 mM **Eu_2_L** at different temperatures. (**b**) Dependence of the chemical shift of the bound water protons on temperature (pH = 7.4, *B*_0_ = 7 T, saturation power *B*_1_= 20 μT, irradiation time 10 s).

Both the chemical shift of the inner-sphere bound water and the saturation efficiency of the bulk water were affected by temperature (Figure 2a). The chemical shift of the bound water pool shifted upfield with an increase in temperature (chemical shift from 57 ppm at 10 °C to 45 ppm at 40 °C). The fitting results suggested a linear-dependence between the chemical shift of the bound-water protons with temperature (Figure 2b). The observed sensitivity of the chemical shift to temperature is 0.4 ppm/°C, which is similar to that of EuDOTAM-Gly [27]. This suggested that **Eu_2_L** could be used to measure temperature distribution in a living subject. The peak width of both bulk and bound water also increased with temperature (Figure 2a), owing to more rapid exchange.

The water exchange rates, *k*_ex_, at different temperatures, were extracted using the quantitative CEST (qCEST) method [28]. In short, the Bloch–McConnell (BM) equations were used for fitting the experimental data, assuming a three-pool fitting model (bulk water, amide protons and the paraCEST pool). The *k*_ex_ values are progressively increasing as the temperature gets higher (Table 1). At 25 °C, the BM fitting three-pool model revealed the exchange rate of around 10 kHz, which corresponds to the bound-water lifetime (τ_M_) of around 100 μs; this value is comparable to τ_M_ reported for EuDOTAM-Gly at same temperature [29]. With such properties displayed, the bismacrocyclic **Eu_2_L** shows very good perspectives for future use as the paraCEST agent. Namely, owing to its bimetallic nature, **Eu_2_L** can produce the CEST signal almost twice stronger than EuDOTAM-Gly, for the same amount of the probe, while potentially having even longer retention time in tissue due to its larger size.

### 2.3. Photophysical Characterization of Eu_2_LTb

**Figure 3:**
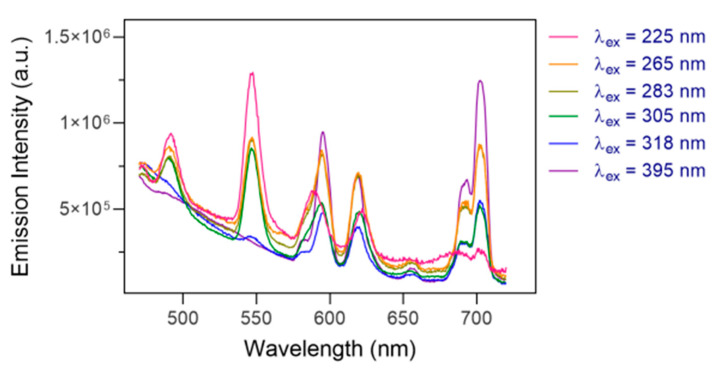
Luminescence spectra of 0.2 mM complex **Eu_2_LTb** at 25 °C, pH 7.4 upon varying the excitation wavelength.

The DA18C6 moiety in complex **Eu_2_L** is also a chelator, albeit capable of binding another type of Ln(III) ion weakly [30,31]. To this end, we investigated the interaction of the **Eu_2_L** with Tb(III) and the photophysical properties of the resulting hetero-trinuclear lanthanide complex **Eu_2_LTb** (Figure S3, Supplementary Materials). This complex displayed mixed emission spectra in solution, which included characteristic peaks of both terbium (^5^D_4_ → ^7^F*_J_*) and europium (^5^D_0_ → ^7^F*_J_*) ions [32]. Specifically, the emission spectra of **Eu_2_LTb** were recorded using excitation wavelengths ranging from 225 nm to 395 nm (Figure 3). The emission excited at 225 nm exhibited the spectrum with four characteristic signals in the visible region at 494 nm (^5^D_4_ → ^7^F_6_), 545 nm (^5^D_4_ → ^7^F_5_), 588 nm (^5^D_4_ → ^7^F_4_), and 625 nm (^5^D_4_ → ^7^F_3_), originating from the Tb(III) ion [33,34]. Concurrently, the emission excited at 395 nm resulted in the spectrum having four characteristic peaks in the visible region at 598 nm, 616 nm, 656 nm, and 702 nm, arising from the ^5^D_0_ → ^7^F*_J_* (J = 1, 2, 3, 4) transitions of the Eu(III) ion, respectively. Additionally, the spectra excited at 265 nm, 283 nm and 305 nm showed the combined emission peaks from both Eu(III) (^5^D_0_ → ^7^F*_J_*) and Tb(III) (^5^D_4_ → ^7^F*_J_*) transitions.

**Figure 4:**
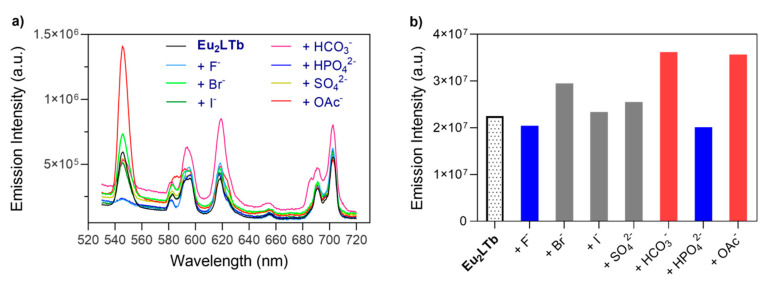
Luminescence selectivity studies of 0.2 mM complex **Eu_2_LTb** at 25 °C, pH 7.4 with various anions (2.0 equiv.), λ_ex_ = 305 nm. (**a**) Emission spectra of **Eu_2_LTb** alone and after addition of F^−^, Br^−^, I^−^, HCO_3_^−^, HPO_4_^2−^, SO_4_^2−^ and OAc^-^. (**b**) Comparison of the recorded luminescence emission intensities monitored in the range between 540 nm and 630 nm.

With the formation of **Eu_2_LTb**, we sought to investigate the potential of this molecule for detection of anions. Namely, the responsiveness of coordinatively unsaturated cyclen-based Ln(III) chelates to small endogenous anions has previously been addressed by many researchers [35,36]. It is well known that the water molecules directly bound to the Eu(III) or Tb(III) ions quench luminescence efficiently due to the high energy of the O-H vibrations [37,38]. On the other hand, anions can occupy the apical position by displacing the water molecule and can circumvent the non-radiative energy quenching, giving rise to an increase in the luminescence intensity. We therefore tested whether the trismacrocyclic host **Eu_2_LTb** can interact with selected biologically important anions. Thus, the interactions between **Eu_2_LTb** and anions including F^−^, Br^−^, I^−^, HCO_3_^−^, HPO_4_^2−^, OAc^−^ and SO_4_^2−^ were assessed, respectively (Figure 4). The solutions were excited at 285 nm in order to cover excitation of both Eu(III) and Tb(III), while the emission intensities were monitored in the range between 540 nm and 630 nm. The results showed no significant change in the emission intensities, except for HCO_3_^−^ and OAc^−^ (Figure 4). Interestingly, HCO_3_^−^ led to emission enhancement of Eu(III), while the OAc^−^ enhanced the luminescence of Tb(III), suggesting that bicarbonates preferentially bind to cyclen-derived chelates, whereas the Tb-18C6 cage dominantly interacts with acetates. Furthermore, anions F^−^ and HPO_4_^2−^ slightly quenched the emission of Tb(III) ion only. It cannot be excluded that the latter is result of the Tb(III)-phosphate formation and precipitation of this salt, which ultimately reduces the Tb(III) centered emission intensity due to elimination of this metal ion from the solution.

## 3. Experimental Section

### 3.1. Materials

Compounds **2** and **5** were synthesized following previously reported procedures [22,24]. All other reagents and solvents were purchased from commercial sources and were used without further purification.

### 3.2. General Methods

Purification of synthesized compounds was performed using silica gel 60 (0.03–0.2 mm) from Carl Roth (Germany). The final ligand and metallated complexes were purified using preparative HPLC on a Varian PrepStar system equipped with the UV−vis detector model 335 and a binary pump model SD-1 manual injector, controlled by Star chromatography workstation version 6.3 software. Low resolution mass spectra were recorded on an ion trap SL 1100 system Agilent with an electrospray ionization source. High resolution mass spectra were recorded on a Bruker Daltonics APEX II (FT-ICR-MS) with an electrospray ionization source.

### 3.3. Synthesis

(2-Bromo-acetylamino)-acetic acid ethyl ester (**2**). A solution of 2-bromoacetyl bromide (17.4 g, 86.0 mmol) in CH_2_Cl_2_ (20 mL) was added dropwise to a stirred cooled solution (0 °C) of the glycine ethyl ester hydrochloride (10.1 g, 72.0 mmol) and K_2_CO_3_ (29.9 g, 216.0 mmol) in a mixture of CH_2_Cl_2_ (100 mL) and water (50 mL). The resulting solution was warmed to room temperature and stirred for 16 h, after which the organic layer was washed with water (2 × 60 mL) and brine (1 × 60 mL), dried over anhydrous Na_2_SO_4_, and concentrated in vacuo. The crude product was recrystallized from EtOAc to afford 2 (12.9 g, 80%) as white crystals [24]. ^1^H-NMR (CDCl_3_, 300 MHz): *δ* (ppm) 1.16–1.47 (m, 3H, C*H*_3_); 3.93 (s, 2H, BrC*H*_2_); 4.06, 4.07 (d, *J* = 5.20 Hz, 2H, NHC*H*_2_); 4.20, 4.23, 4.25, 4.28 (q, *J* = 7.18 Hz, 2H, OC*H*_2_). ^13^C-NMR (CDCl_3_, 75 MHz): *δ* (ppm) 13.9 (*C*H_3_); 32.0 (Br*C*H_2_); 42.0 (NH*C*H_2_); 61.7 (O*C*H_2_CH_3_); 168.7 (*C*ONH); 170.4 (*C*OO).

{4,7,10-Tris-[(ethoxycarbonylmethyl-carbamoyl)-methyl]-1,4,7,10tetraaza-cyclododec-1-yl}-acetic acid benzyl ester (**3**). The mixture of cyclen-monoacetate benzyl ester **1** (3.2 g, 10.0 mmol) and Na_2_CO_3_ (4.2 g, 40.0 mmol) were stirred in CH_2_Cl_2_ (35 mL) at room temperature for 10 min, then compound **2** in CH_2_Cl_2_ (9.0 g, 40.0 mmol in 15 mL) was added dropwise. The reaction mixture was stirred at room temperature for 12 h. Upon reaction completion, the reaction mixture was filtered off, the filtrate was evaporated, and the solid residue was purified by silica column chromatography using CH_2_Cl_2_/MeOH (*v/v*, 20:1) as the eluent to yield 2 (3.8 g, 50%) as a yellow oil. ^1^H-NMR (CDCl_3_, 300 MHz): *δ* (ppm): 1.14–1.31 (m, 9H, C*H*_3_); 2.16–2.90 (br, 16H, NC*H*_2_CH_2_); 3.05–3.54 (br, 8H, NC*H*_2_CO); 3.87–4.26 (br, 12H, NHC*H*_2_C, OC*H*_2_CH_3_); 5.14 (s, 2H, ArC*H*_2_O); 7.28–7.46 (m, 5H, Ar*H*). ^13^C-NMR (CDCl_3_, 75 MHz): *δ* (ppm): 14.3 (*C*H_3_); 41.0, 41.1, 41.3 (NH*C*H_2_); 50.6, 55.6, 57.2, 57.5, 58.3 (N*C*H_2_); 61.4 (O*C*H_2_CH_3_); 67.0 (Ar*C*H_2_); 128.5, 128.8 (Ar*C*H); 135.5 (Ar*C*CH_2_); 170.0, 170.2 (*C*ONH); 172.5, 172.8 (*C*OO); ESI-HRMS: (*m/z*) [M + H]^+^ calcd for C_35_H_56_N_7_O_11_^+^, 750.4032; found: 750.4031.

{4,7,10-Tris-[(ethoxycarbonylmethyl-carbamoyl)-methyl]-1,4,7,10tetraaza-cyclododec-1-yl}-acetic acid (**4**). The compound **3** (2.0 g, 2.7 mmol) was dissolved in DMF (25 mL). The catalyst Pd/C (10%, *w/w*, 0.2 equiv) and 10 µL of ammonia in MeOH (7 M) were added. The mixture solution was shaken in Parr apparatus in the atmosphere of H_2_ (3.2 bar) for 12 h at room temperature. The resulting solution was filtered off and concentrated in vacuo to afford 4 (1.6 g, 90%) as a brown oil. ^1^H-NMR (CDCl_3_, 300 MHz): *δ* (ppm): 1.14–1.36 (br, 9H, C*H*_3_); 2.14–3.78 (br, 24H, NC*H*_2_); 3.79–4.26 (br, 12H, NHC*H*_2_, OC*H*_2_). ^13^C-NMR (CDCl_3_, 75 MHz): *δ* (ppm): 14.1 (*C*H_3_); 41.2 (NH*C*H_2_); 49.9, 50.5, 57.6, 57.7(N*C*H_2_); 61.4 (O*C*H_2_CH_3_); 170.0, 170.1 (*C*ONH); 172.1 (*C*OO); ESI-HRMS: (*m/z*) [M + H]^+^ calcd for C_28_H_50_N_7_O_11_^+^, 660.3563; found: 660.3559.

(2-Bromo-ethyl)-carbamic acid benzyl ester (**5**). To a mixture of 2-bromoethylamine hydrogen bromide (2.0 g, 10.0 mmol) and triethylamine Et_3_N (3.0 g, 30.0 mmol) in CH_2_Cl_2_ (40 mL), benzyl chloroformate (2.7 g, 16.0 mmol) was added in a portion-wise manner at 0 °C. The resulting mixture was stirred at 0 °C for 6 h. The mixture was then washed with aqueous NaHCO_3_ (10%, 3 × 10 mL) and citric acid (10%, 3 × 10 mL) and dried over anhydrous Na_2_SO_4_. The solvent was then evaporated to obtain crude residue, which was purified by column chromatography (silica gel, hexane/EtOAc, 7:1) to afford 5 (2.3 g, 90 % yield) as a white crystal solid [22]. ^1^H-NMR (CDCl_3_, 300 MHz): *δ* (ppm): 3.01–3.56 (m, 4H, BrC*H*_2_, C*H*_2_NH); 5.04 (s, 2H, ArC*H*_2_); 7.21–7.36 (br, 5H, Ar*H*). ^13^C-NMR (CDCl_3_, 75 MHz): *δ* (ppm): 31.8 (Br*C*H_2_); 42.4 (*C*H_2_NH); 66.5 (Ar*C*H_2_); 126.6, 127.7, 128.2 (Ar*C*H); 136.0 (Ar*C*CH_2_); 155.3 (*C*ONH).

{2-[16-(2-Benzyloxycarbonylamino-ethyl)-1,4,10,13-tetraoxa-7,16-diaza-cyclooctadec-7-yl]-ethyl}-carbamic acid benzyl ester (**6**). Cs_2_CO_3_ (3.9 g, 12.0 mmol) was added to a solution of 1,10-diaza-18-crown-6 (0.8 g, 3.0 mmol) in dry MeCN (15 mL). The obtained suspension was stirred for 10 min at room temperature and then **5** (1.7 g, 6.6 mmol) was added to it. The mixture was heated to 65 °C and stirred for 1.5 h after which additional amount of **5** (0.8 g, 3.1 mmol) was added and the reaction mixture was stirred for 3 h. Afterwards, the solvent was evaporated under the reduced pressure and the crude was purified by column chromatography (silica gel, CH_2_Cl_2_/MeOH, 100:7) to give **6** (1.6 g, 58%) as a brown oil. ^1^H-NMR (CDCl_3_, 300 MHz): *δ* (ppm): 2.43–2.98 (br, 12H, NC*H*_2_CH_2_); 3.17–3.39 (br, 4H, CH_2_C*H*_2_NH); 3.47–3.64 (br, 16H, C*H*_2_OC*H*_2_); 5.08 (s, 2H, ArC*H*_2_); 7.21–7.45 (br, 10H, Ar*H*). ^13^C-NMR (CDCl_3_, 75 MHz): *δ* (ppm): 38.9 (*C*H_2_NH); 53.5 (N*C*H_2_CH_2_); 66.3 (Ar*C*H_2_); 67.6, 68.7 (*C*H_2_O*C*H_2_); 127.8, 128.2, 128.5 (Ar*C*H); 136.6 (Ar*C*CH_2_); 156.9 (*C*ONH). ESI-HRMS: (*m/z*) [M + Na]^+^ calcd for C_32_H_48_N_4_NaO_8_^+^, 639.3364; found: 639.3376.

2-[16-(2-Amino-ethyl)-1,4,10,13-tetraoxa-7,16-diaza-cyclooctadec-7-yl]-ethylamine (**7**). The compound **6** (1.2 g, 1.9 mmol) was dissolved in EtOH (25 mL). The catalyst Pd/C (10% *w/w*, 0.2 equiv.) and 10 µL of ammonia in MeOH (7 M) were added. The solution was shaken in Parr apparatus in the atmosphere of H_2_ (3.2 bar) for 4 h at room temperature. Removal of the catalyst by filtration and evaporation of EtOH yielded the diamine **7** (0.6 g, 88%). ^1^H-NMR (D_2_O, 300 MHz): *δ* (ppm): 2.68–2.94 (br, 12H, NC*H*_2_CH_2_); 3.28–3.33 (br, 4H, CH_2_C*H*_2_NH_2_); 3.48–3.81 (br, 16H, C*H*_2_OC*H*_2_). ^13^C-NMR (D_2_O, 75 MHz): *δ* (ppm): 36.9 (*C*H_2_NH_2_); 51.8, 53.9 (N*C*H_2_CH_2_); 68.2, 69.7 (*C*H_2_O*C*H_2_). ESI-HRMS: (*m/z*) [M + H]^+^ calcd for C_16_H_37_N_4_O_4_^+^, 349.2809; found: 349.2816.

Compound **8**. The acid **4** (1.1 g, 1.7 mmol) and HATU (1-[Bis(dimethylamino)methylene]-1H-1,2,3-triazolo[4,5-b]pyridinium 3-oxide hexafluorophosphate, Hexafluorophosphate Azabenzotriazole Tetramethyl Uronium, 0.7 g, 1.9 mmol) were subsequently added to a stirred solution of diamine **7** (0.2 g, 0.6 mmol) in dry DMF (10 mL), after which the reaction mixture was stirred for 6 h at room temperature. The solvent was removed under the reduced pressure and the residue was washed with Et_2_O (3 × 10 mL), CH_2_Cl_2_ (3 × 10 mL) and MeOH (2 × 10 mL). The collected filtrate was concentrated in vacuo to give a yellowish oil (0.4 g, 48%). ^1^H-NMR (CD_3_OD, 300 MHz): *δ* (ppm): 1.23, 1.25, 1.28 (t, *J* = 7.50 Hz, 18H, C*H*_3_); 3.39–3.83 (br, 64H, C*H*_2_); 3.94–4.05 (br, 16H, OC*H*_2_); 4.05–4.12 (br, 12H, NHC*H*_2_C); 4.14–4.26 (m, 12H, NHC*H*_2_CO). ^13^C-NMR (CD_3_OD, 75 MHz): *δ* (ppm): 14.5 (*C*H_3_); 42.2 (NH*C*H_2_); 55.2, 56.3, 62.0, 64.7, 65.3, 70.9 (N*C*H_2_); 170.3, (*C*ONH, *C*OO). ESI-HRMS: (*m/z*) [M + 3H]^3+^ calcd for C_72_H_133_N_18_O_24_^3+^, 544.6575; found: 544.6575.

Compound **H_6_L.** Compound **8** (300 mg, 0.2 mmol) was dissolved in MeOH (5 mL) and treated with LiOH (130 mg, 5.5 mmol) at room temperature for 12 h. After removal of LiOH by filtration, MeOH was evaporated. The crude mixture was dissolved in water and the pH was adjusted to 7 and purified by preparative HPLC. The white powder **H_6_L** (120 mg, 45%) was obtained by lyophilization. ^1^H-NMR (D_2_O, 300 MHz): δ (ppm): 3.04–3.31 (br, 30H, cyclen NCH_2_), 3.33–3.52 (br, 18H, cyclen NCH_2_), 3.53–3.62 (br, 12H, crown ether NCH_2_), 3.63–3.79 (br, 16H, crown ether OCH_2_), 3.80–3.96 (br, 16H, CONHCH_2_). ^13^C-NMR (D_2_O, 75 MHz): δ (ppm): 41.1 (NH*C*H_2_); 53.2, 54.8, 55.1, 63.6 (N*C*H_2_); 69.7 (O*C*H_2_); 162.8, 172.8, (*C*ONH, *C*OOH). ESI-HRMS: (*m/z*) [M − H]^−^ calcd for C_60_H_105_N_18_O_24_^-^, 1461.7555; found: 1461.7548.

Complex **Eu_2_L.** The ligand **H_6_L** (100 mg, 0.07 mmol) was dissolved in MilliQ water and the pH value was set to 7. The aqueous solution of EuCl_3_∙6H_2_O (55 mg, 0.15 mmol) was added dropwise to the ligand solution. The mixture was heated at 50 °C for 12 h and the pH of the solution was periodically adjusted to 7.0 by addition of 0.1 M NaOH solution. Then, the reaction mixture was cooled to room temperature and purified by HPLC. The yellow solid compound (84 mg, 70%) was obtained by lyophilization. ESI-HRMS: (*m/z*) [M + 3H]^3+^ calcd for C_60_H_103_Eu_2_N_18_O_24_^3+^, 588.5267; found: 588.5279.

Complex **Eu_2_LTb.** To a stirred solution of **Eu_2_L** (40 mg, 0.02 mmol) in MilliQ water (pH~7), the aqueous solution of TbCl_3_∙6H_2_O (10 mg, 0.03 mmol) was added dropwise while maintaining pH with 0.1 M NaOH (aq). The reaction mixture was stirred at 50 °C for 6 h. Then the solvent was reduced under the vacuum and the gray solid powder was obtained upon lyophilization. LC-MS: (*m/z*) [M + H]^2+^ calcd for C_60_H_99_Eu_2_N_18_O_24_Tb^2+^, 959.3; found: 959.3.

### 3.4. NMR Spectroscopy

^1^H, ^13^C-NMR and CEST experiments were recorded on a Bruker Avance III 300 MHz spectrometer. ^1^H and ^13^C-NMR spectra were recorded at 25 °C, using either CDCl_3_ or D_2_O and referenced to TMS/TSP. Processing was performed using TopSpin 2.1 (Bruker GmbH) and ACD/SpecManager 9.0 (Advanced Chemistry Development, Inc., Toronto, Canada). The concentrations of **Eu_2_L** and TbCl_3_ were determined using the bulk magnetic susceptibility shift (BMS) method [39]. CEST spectra were obtained in 10% D_2_O and 90% H_2_O solutions of the paramagnetic complex, using a saturation time of 10 s at 7 T and different temperatures (10, 15, 20, 25, 30, 35, 37 and 40 °C) and a frequency-offset range of ± 100 ppm with 1 ppm resolution. The longitudinal and transverse relaxation times, *T*_1_ and *T*_2_, were measured using the inversion-recovery and Carr–Purcell–Meiboom–Gill pulse sequences, respectively [40,41].

### 3.5. Optical Spectroscopy

All fluorescence spectra were recorded on a QuantaMaster^TM^ 3 PH fluorescence spectrometer from Photon Technology International, Inc. (USA) at 25 °C and pH 7.4. Titration experiments with Tb(III) were performed by following the emission intensity at 545 nm. For the anion selectivity experiments, appropriate concentration (0.2 mM) of F^−^, Br^−^, I^−^, HCO_3_^−^, SO_4_^2−^, OAc^−^ and HPO_4_^2−^ were prepared by dilution method using HPLC grade water. All data were recorded in HEPES buffer (50 mM, pH 7.4), using the excitation wavelength at 305 nm and the slit widths of 5 nm and 1 nm for excitation and emission, respectively.

## 4. Conclusions

A trismacrocyclic DOTA-amide DA18C6-based ligand framework was prepared and characterized. The novel dinuclear **Eu_2_L** contrast agent showed the paraCEST properties typical to that of well-known EuDOTAM-Gly probe, albeit with greater potential for stronger CEST effect due to its bismacrocyclic and dinuclear nature. Upon introduction of Tb(III) ions, the resulting trinuclear **Eu_2_LTb** complex exhibited mixed luminescence emission. Moreover, the binding studies with various anions revealed specificity to HCO_3_^−^ and OAc^−^ over other biologically relevant anions. The typical paraCEST signal and combined luminescence emission properties pave the way for this class of mixed macrocyclic ligands to develop further as potential dual-modal MRI/luminescence probes.

## Figures and Tables

**Table 1:** The *k_ex_* values of bound water molecule (5 mM complex **Eu_2_L**) using the quantitative CEST (qCEST) method at different temperatures. Each temperature experiment was recorded at pH 7.4, with irradiation time of 10 s and different *B*_1_ (2.5, 5, 10, 15, 20, 25 and 30 μT).

Temp./°C	10	15	20	25	30	35	37	40
*k_ex_*/kHz	3.8 ± 0.4	4.7 ± 0.3	6.3 ± 0.3	8.9 ± 0.3	12.9 ± 0.4	18.6 ± 0.5	21.5 ± 0.5	26.7 ± 0.6

## Supplementary Materials

The supplementary materials are available online. Figure S1: Change in T1 and T2 relaxation times for 5 mM **Eu_2_L** with temperature at 300 MHz (50 mM HEPES, pH 7.4), Figure S2: The CEST spectra of 5 mM **Eu_2_L** at different temperatures and saturation power B1, Figure S3: Emission intensity monitored at 545 nm of 0.2 mM **Eu_2_L** upon titration with Tb^3+^ at 25 °C (50 mM HEPES, pH 7.4).
