# Supplementary material for: Macrocyclic Chelates Bridged by a Diaza-Crown Ether: Towards Multinuclear Bimodal Molecular Imaging Probes
Source: Molecules. 2020 Oct 29;25(21):5019. doi: 10.3390/molecules25215019 (PMC7663075; doi:10.3390/molecules25215019)

Supporting Information for

Macrocyclic Chelates Bridged by a Diaza-crown Ether: Towards Multinuclear Bimodal Molecular Imaging Probes

Gaoji Wang[1] and Goran Angelovski[1,2*]

[1] MR Neuroimaging Agents, MPI for Biological Cybernetics, Tübingen, Germany.
[2] Lab of Molecular and Cellular Neuroimaging, International Center for Primate Brain Research (ICPBR), Center for Excellence in Brain Science and Intelligence Technology (CEBSIT), Chinese Academy of Science (CAS), Shanghai 200031, PR China

*E-mail: goran.angelovski@tuebingen.mpg.de

Contents

NMR CEST experiments S2
Luminescence experiments S4
NMR spectra S5

NMR CEST experiments

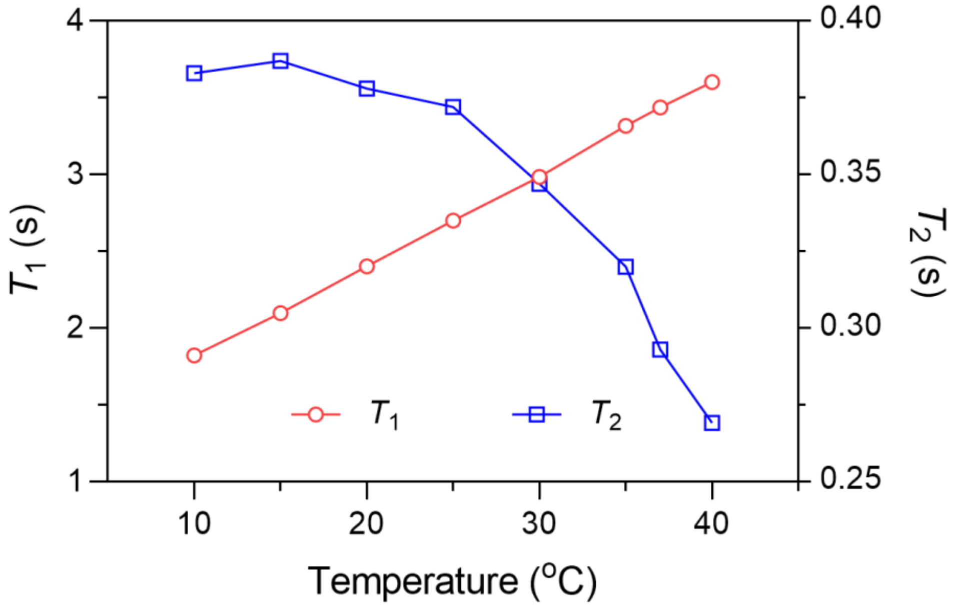

Figure S1. Change in T_1 and T_2 relaxation times for 5 mM **Eu_2L** with temperature at 300 MHz (50 mM HEPES, pH 7.4).

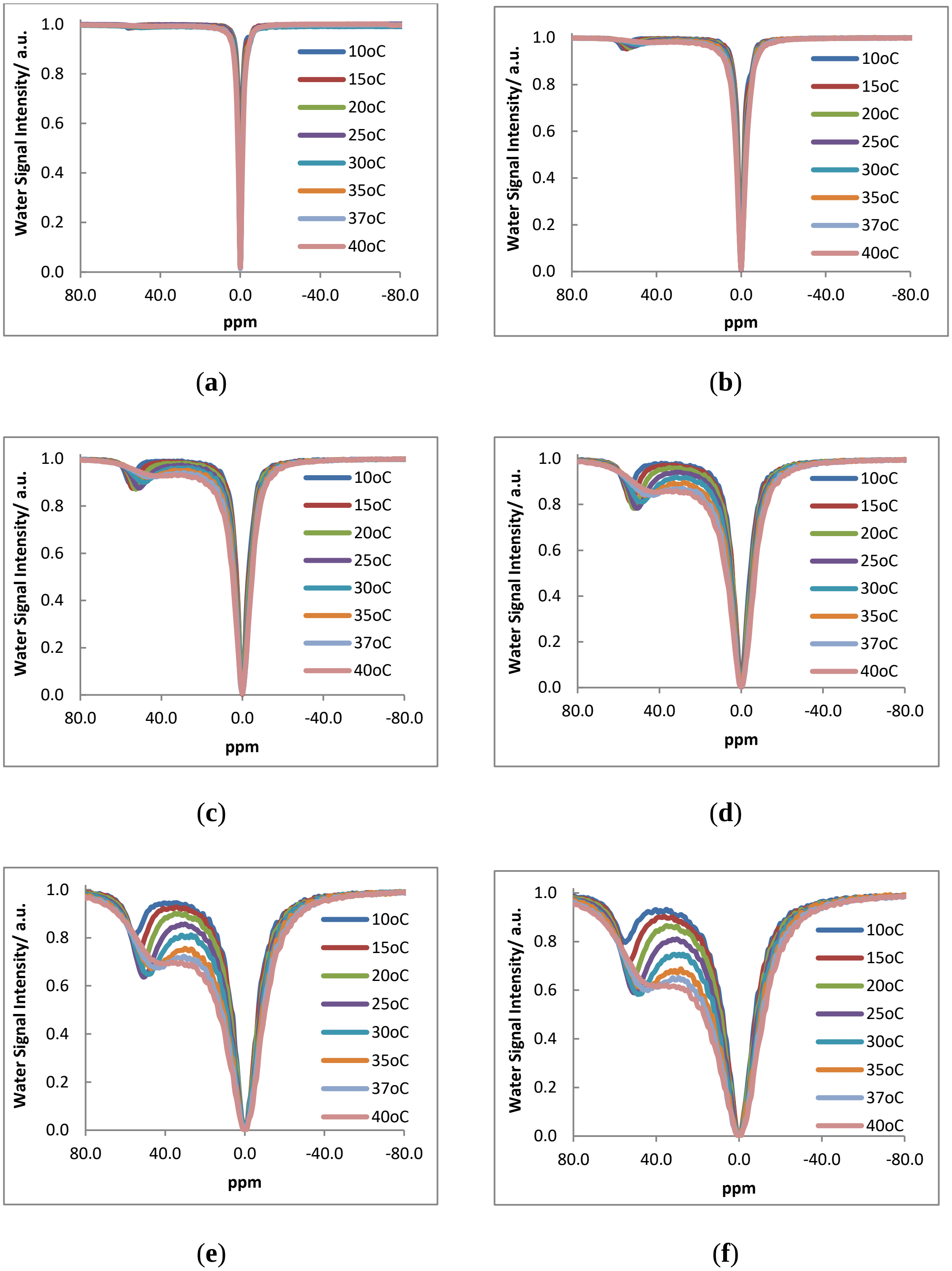

Figure S2. The CEST spectra of 5 mM **Eu$_2$L** at different temperatures and saturation power B_1: (a) 2.5 μT, (b) 5.0 μT, (c) 10 μT, (d) 15 μT, (e) 25 μT and (f) 30 μT.

Luminescence experiments

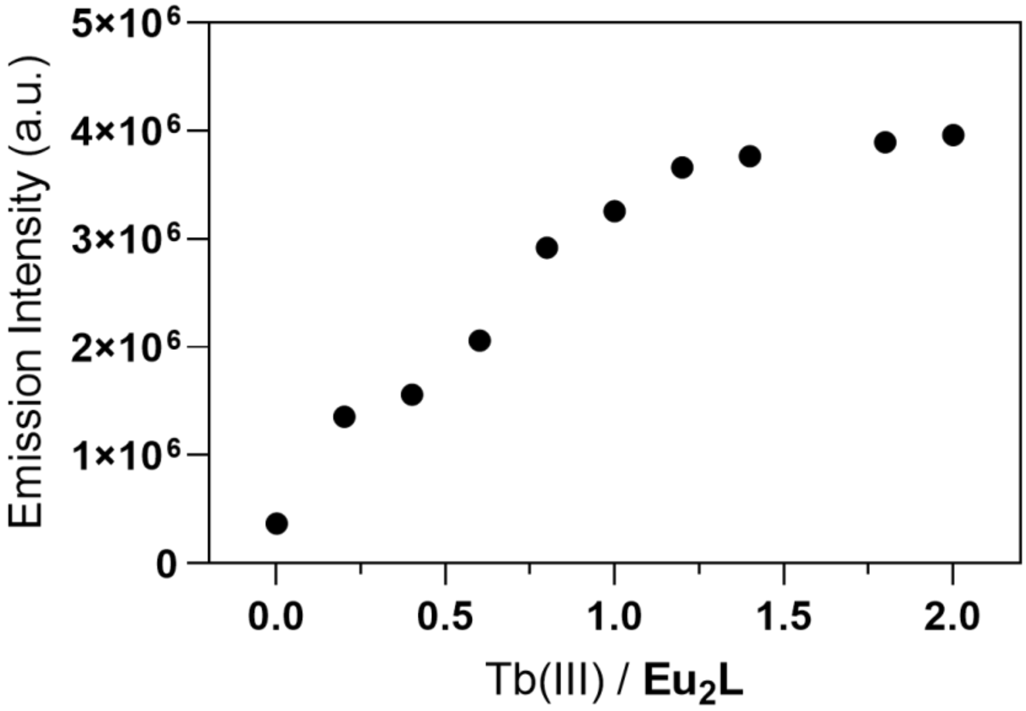

Figure S3. Emission intensity monitored at 545 nm of 0.2 mM **Eu_2L** upon titration with Tb^{3+} at 25 °C (50 mM HEPES, pH 7.4). The binding isotherm saturates after 1 equiv. of added Tb^{3+}, indicating formation of weak 1:1 complex between **Eu_2L** and Tb^{3+}.

NMR spectra

Compound **2**

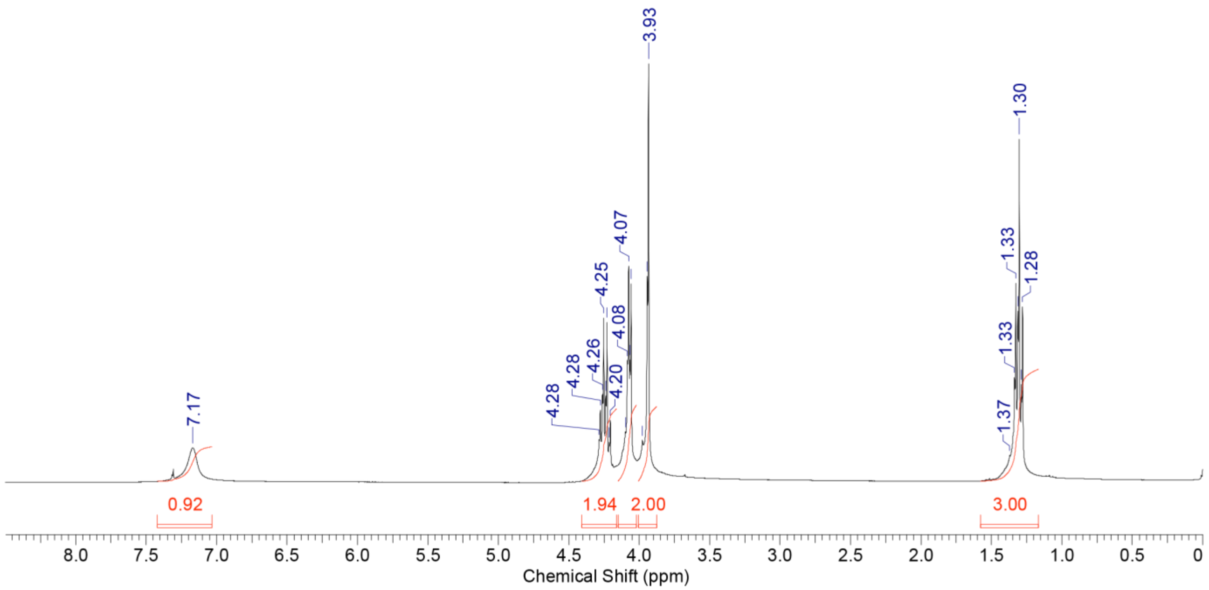

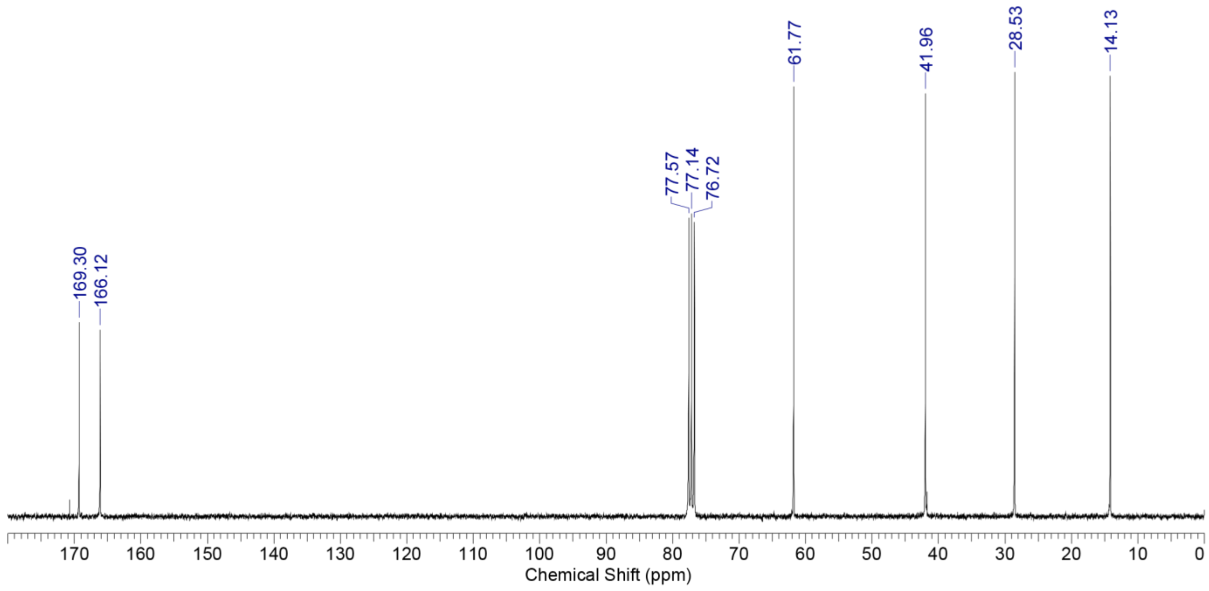

Compound **3**

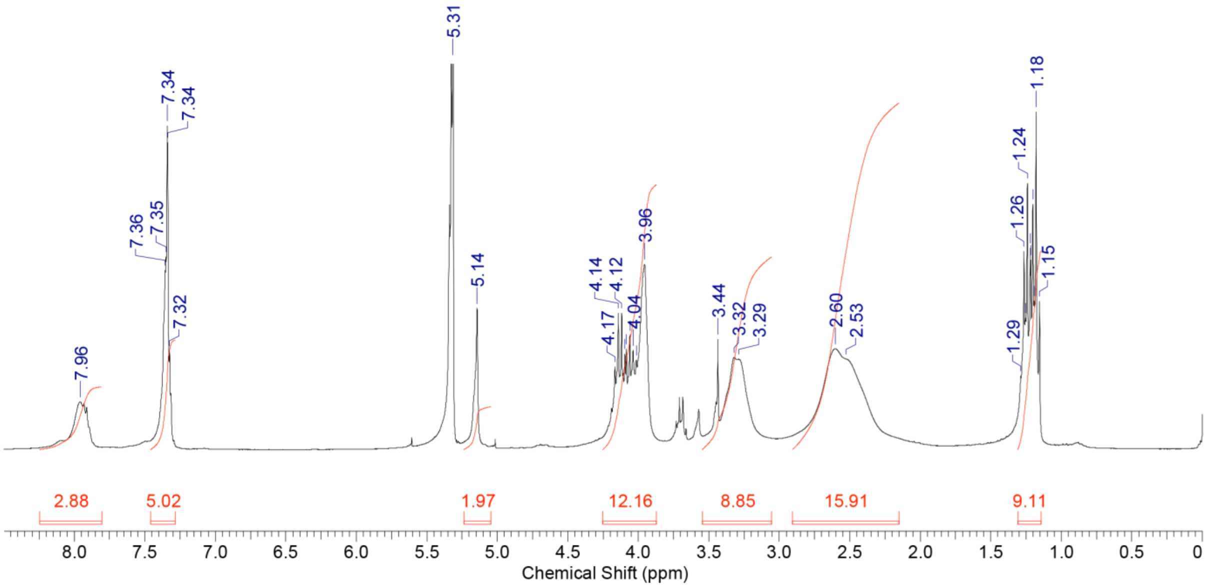
5.31
7.34
7.34
7.36
7.35
7.32
7.96
5.14
4.14
4.12
4.17
4.04
3.96
3.44
3.32
3.29
2.60
2.53
1.18
1.24
1.26
1.15
1.29
2.88
5.02
1.97
12.16
8.85
15.91
9.11
Chemical Shift (ppm)

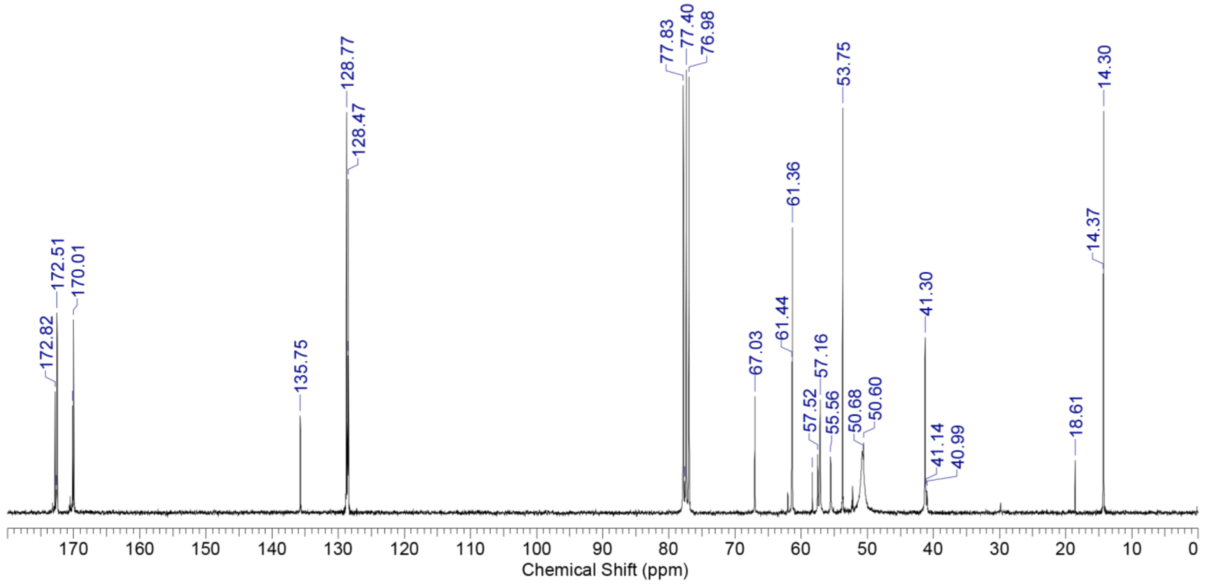
172.82
172.51
170.01
135.75
128.77
128.47
77.83
77.40
76.98
67.03
61.44
61.36
57.52
57.16
55.56
53.75
50.68
50.60
41.30
41.14
40.99
18.61
14.37
14.30
Chemical Shift (ppm)

Compound **4**

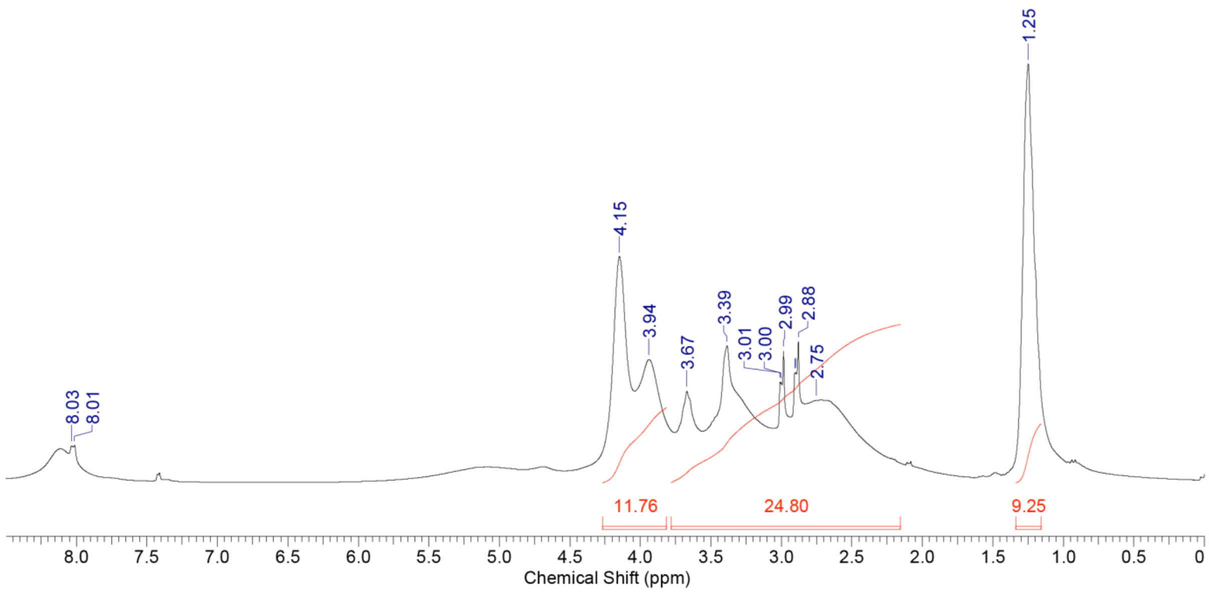
4.15
3.94
3.67
3.39
3.01
3.00
2.99
2.88
2.75
8.03
8.01
1.25
11.76
24.80
9.25
Chemical Shift (ppm)

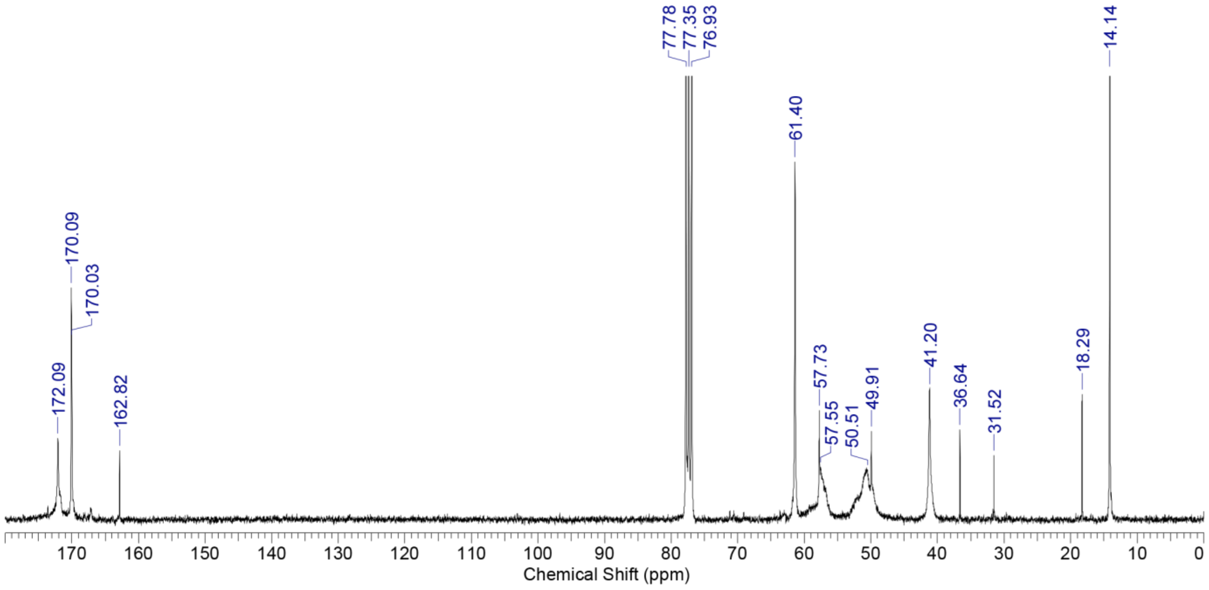
77.78
77.35
76.93
61.40
14.14
170.09
170.03
172.09
162.82
57.73
57.55
50.51
49.91
41.20
36.64
31.52
18.29
Chemical Shift (ppm)

Compound **5**

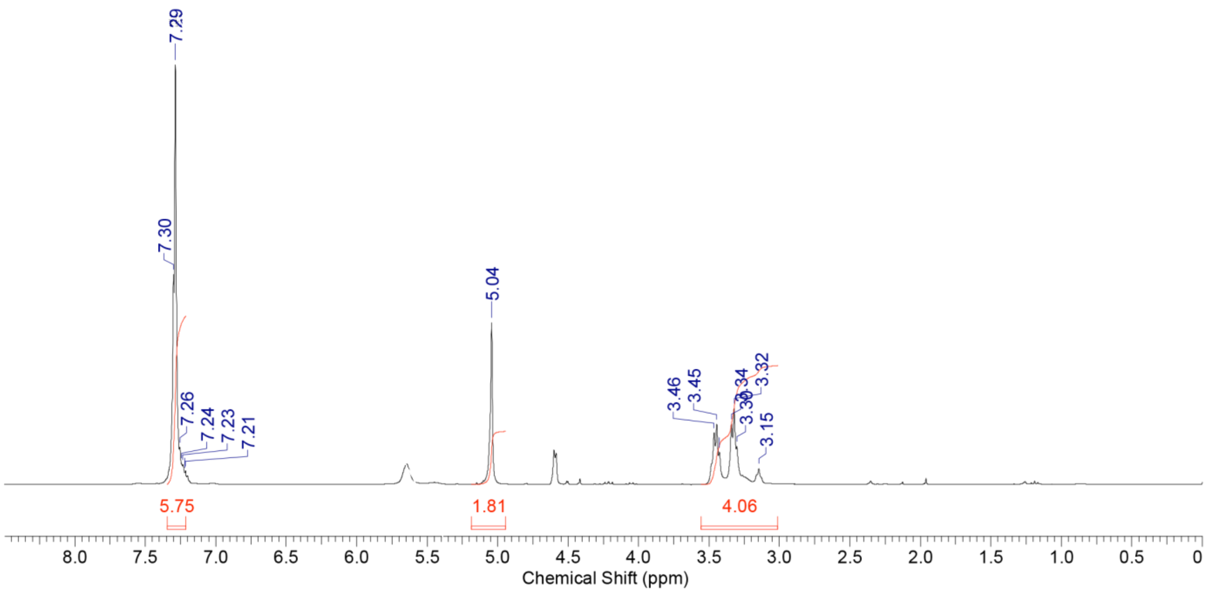
7.29
7.30
7.26
7.24
7.23
7.21
5.04
3.46
3.45
3.34
3.32
3.30
3.15
5.75
1.81
4.06
8.0 7.5 7.0 6.5 6.0 5.5 5.0 4.5 4.0 3.5 3.0 2.5 2.0 1.5 1.0 0.5 0
Chemical Shift (ppm)

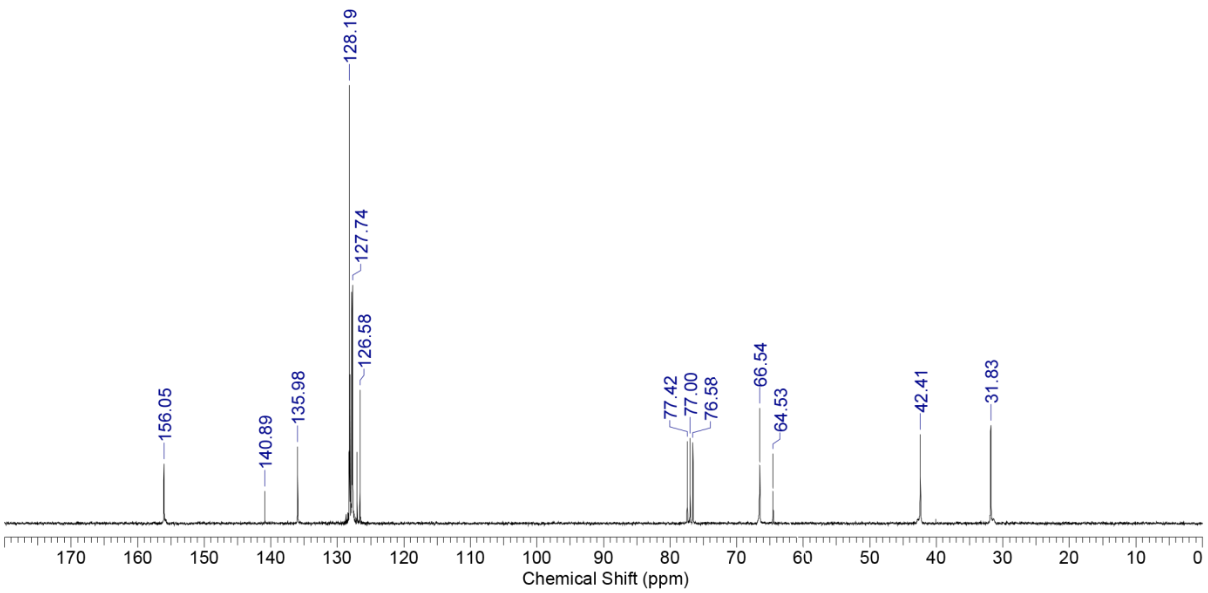
128.19
127.74
126.58
156.05
140.89
135.98
77.42
77.00
76.58
66.54
64.53
42.41
31.83
170 160 150 140 130 120 110 100 90 80 70 60 50 40 30 20 10 0
Chemical Shift (ppm)

Compound **6**

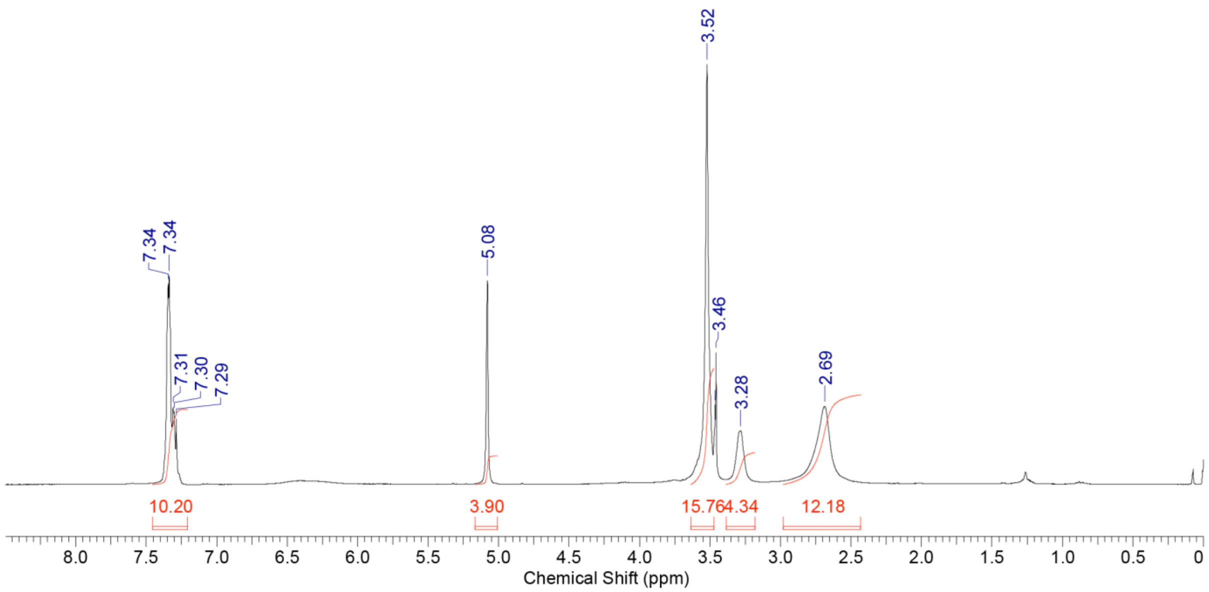

7.34
7.34
7.31
7.30
7.29
5.08
3.52
3.46
3.28
2.69
10.20
3.90
15.76
4.34
12.18
8.0 7.5 7.0 6.5 6.0 5.5 5.0 4.5 4.0 3.5 3.0 2.5 2.0 1.5 1.0 0.5 0
Chemical Shift (ppm)

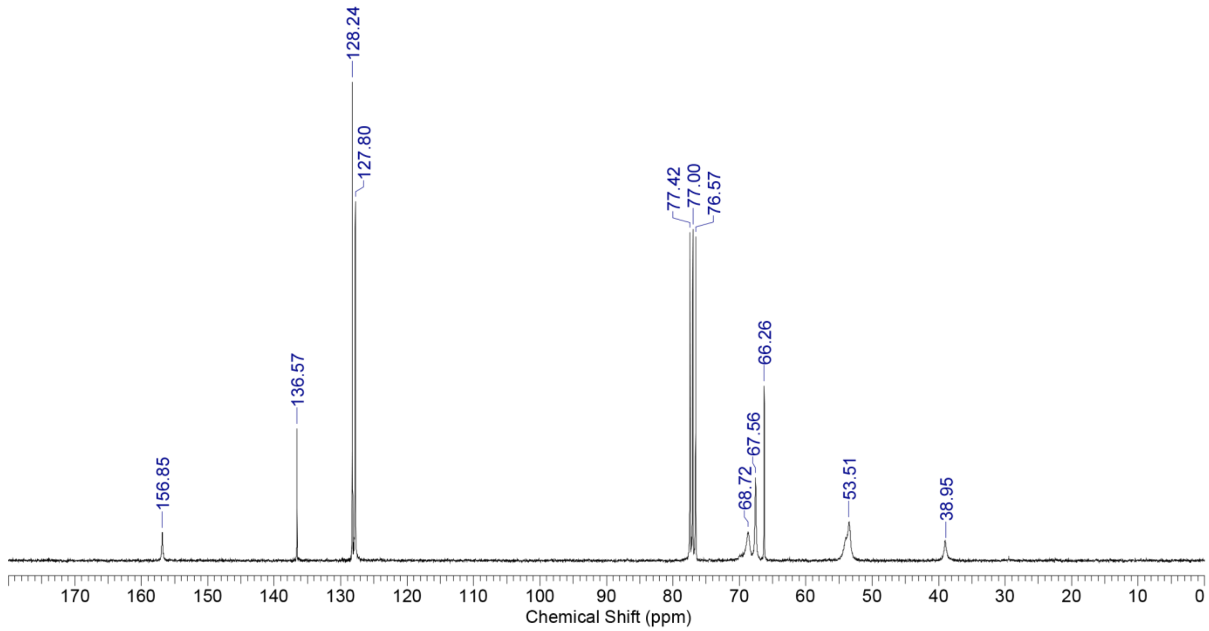

156.85
136.57
128.24
127.80
77.42
77.00
76.57
68.72
67.56
66.26
53.51
38.95
170 160 150 140 130 120 110 100 90 80 70 60 50 40 30 20 10 0
Chemical Shift (ppm)

Compound **7**

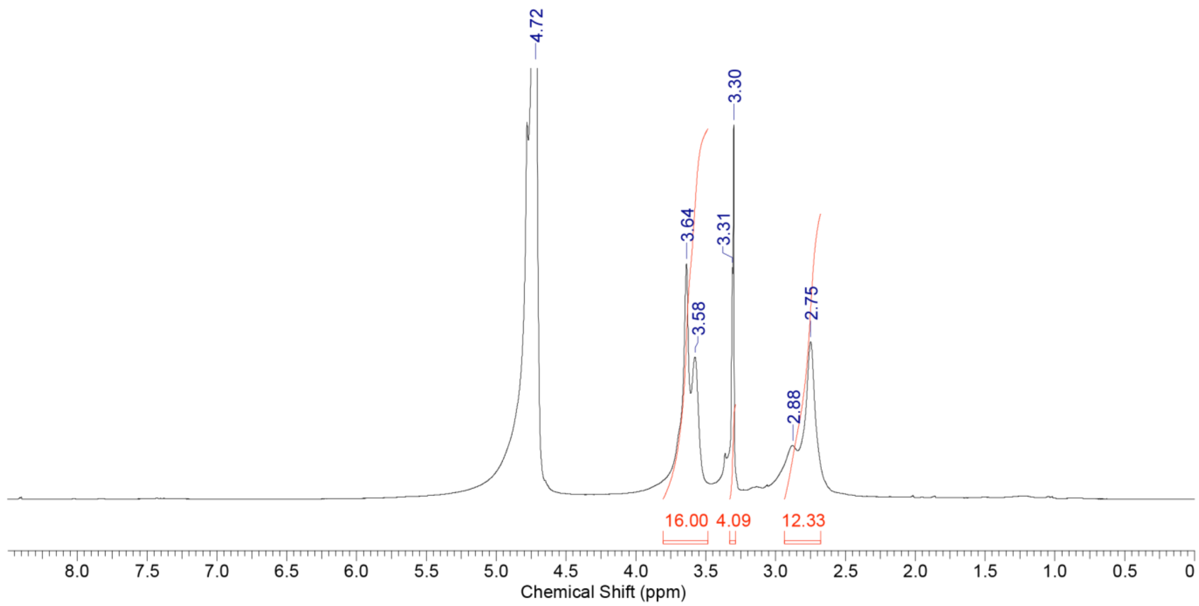
4.72
3.30
3.64
3.31
3.58
2.75
2.88
16.00
4.09
12.33
8.0
7.5
7.0
6.5
6.0
5.5
5.0
4.5
4.0
3.5
3.0
2.5
2.0
1.5
1.0
0.5
0
Chemical Shift (ppm)

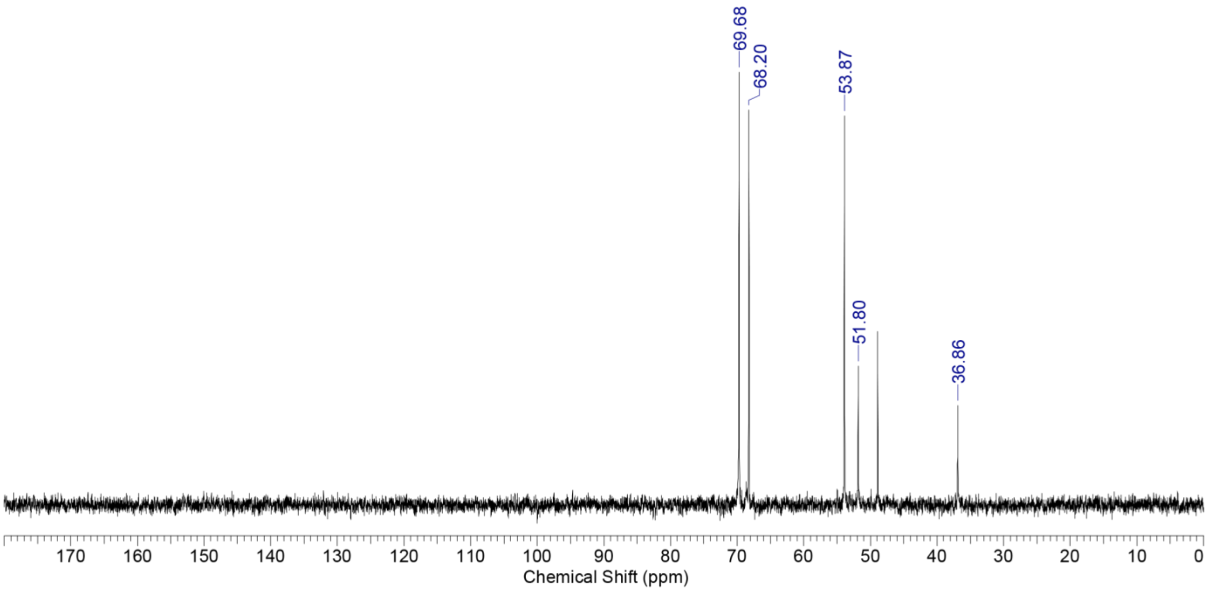
69.68
68.20
53.87
51.80
36.86
170
160
150
140
130
120
110
100
90
80
70
60
50
40
30
20
10
0
Chemical Shift (ppm)

Compound **8**

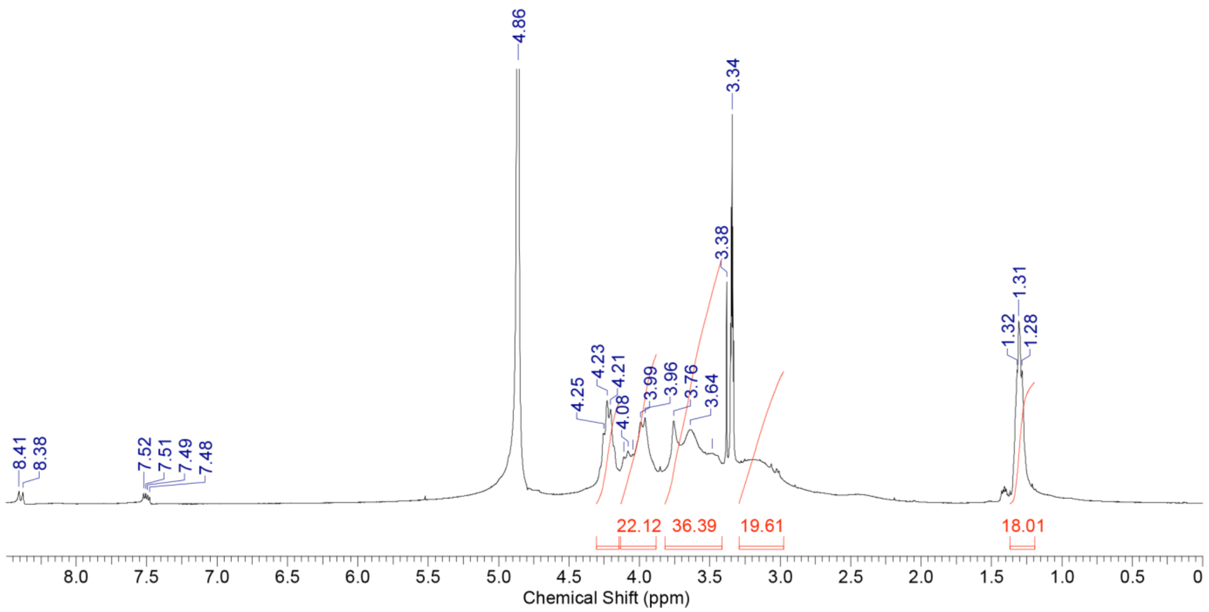

4.86
3.34
3.38
1.31
1.32
1.28
4.23
4.21
4.25
4.08
3.99
3.96
3.76
3.64
8.41
8.38
7.52
7.51
7.49
7.48
22.12
36.39
19.61
18.01
8.0
7.5
7.0
6.5
6.0
5.5
5.0
4.5
4.0
3.5
3.0
2.5
2.0
1.5
1.0
0.5
0
Chemical Shift (ppm)

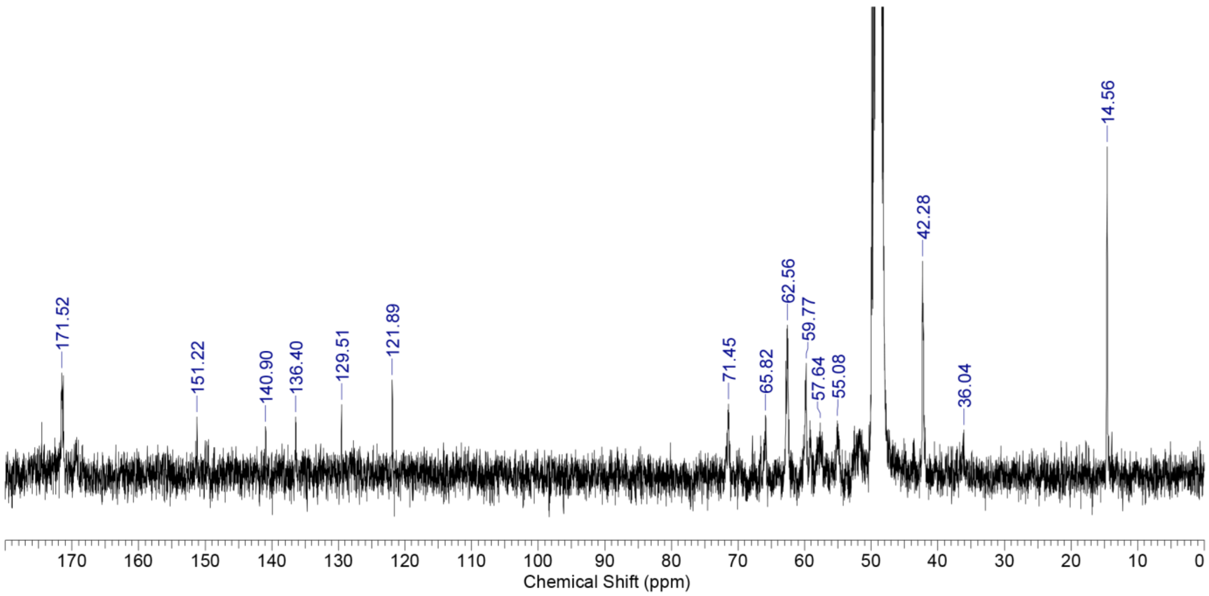

14.56
42.28
62.56
59.77
171.52
121.89
129.51
151.22
140.90
136.40
71.45
65.82
57.64
55.08
36.04
170
160
150
140
130
120
110
100
90
80
70
60
50
40
30
20
10
0
Chemical Shift (ppm)

Compound **H_6L**

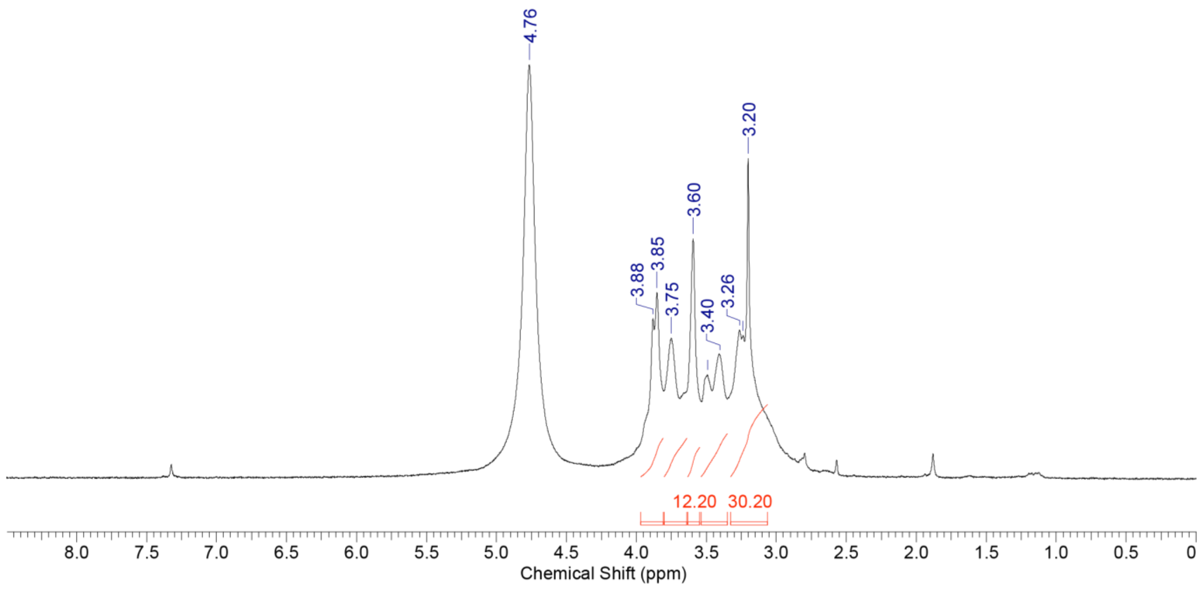

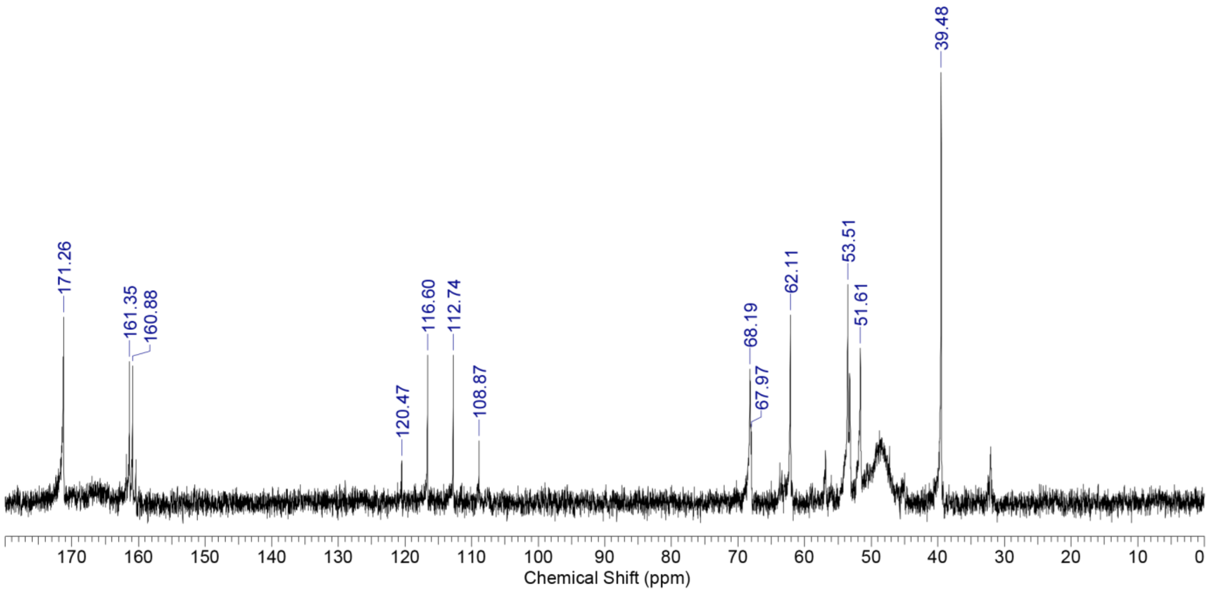

Supplement: Supplementary file 1 [file molecules-25-05019-s001.pdf]
